# Exosomal circular RNA: a signature for lung cancer progression

**DOI:** 10.1186/s12935-022-02793-7

**Published:** 2022-12-01

**Authors:** Bashdar Mahmud Hussen, Snur Rasool Abdullah, Goran Sedeeq Hama Faraj, Mohammed Fatih Rasul, Abbas Salihi, Soudeh Ghafouri-Fard, Mohammad Taheri, Majid Mokhtari

**Affiliations:** 1 Department of Pharmacognosy, College of Pharmacy, Hawler Medical University, Erbil, Kurdistan Region Iraq; 2 Medical Laboratory Science, Lebanese French University, Erbil, Kurdistan Region Iraq; 3 Department of Medical Laboratory Science, Komar University of Science and Technology, Sulaymaniyah, Iraq; 4 Department of Pharmaceutical Basic Science, Faculty of Pharmacy, Tishk International University, Erbil, Kurdistan Region Iraq; 5 Department of Biology, College of Science, Salahaddin University-Erbil, Erbil, Kurdistan Region Iraq; 6 Department of Biomedical Sciences, Cihan University-Erbil, Kurdistan Region, Erbil, 44001, Iraq; 7 Department of Medical Genetics, Shahid Beheshti University of Medical Sciences, Tehran, Iran; 8 Urology and Nephrology Research Center, Shahid Beheshti University of Medical Sciences, Tehran, Iran; 9 Institute of Human Genetics, Jena University Hospital, Jena, Germany; 10 Tracheal Diseases Research Center, National Research Institute of Tuberculosis and Lung Diseases (NRITLD), Shahid Beheshti University of Medical Sciences, Tehran, Iran

**Keywords:** Lung Cancer (LC), Circular RNA (circRNA), Exosomal circular RNA (exo-circRNA)

## Abstract

Membrane vesicles having a diameter of 30–150 nm are known as exosomes. Several cancer types secrete exosomes, which may contain proteins, circular RNAs (circRNAs), microRNAs, or DNA. CircRNAs are endogenous RNAs that do not code for proteins and can create continuous and covalently closed loops. In cancer pathogenesis, especially metastasis, exosomal circRNAs (exo-circRNAs) have a crucial role mainly due to the frequently aberrant expression levels within tumors. However, neither the activities nor the regulatory mechanisms of exo-circRNAs in advancing lung cancer (LC) are obvious. A better understanding of the regulation and network connections of exo-circRNAs will lead to better treatment for LCs. The main objective of the current review is to highlight the functions and mechanisms of exo-circRNAs in LC and assess the relationships between exo-circRNA dysregulation and LC progression. In addition, underline the possible therapeutic targets based on exo-circRNA modulating.

## Introduction

Lung cancer (LC) is the most frequent type of cancer worldwide and the leading cause of cancer mortality [1]. An essential factor in LC deaths is the invasion and metastasis of cancer cells through the circulation or lymphatic systems, Which is a significant cause of mortality in patients [2]. Tumor-derived exosomes (TDEs) play a vital function in the tumor microenvironment by facilitating the development of a pre-metastatic niche [3]. Exosomes are small membrane vesicles with a diameter of 30–150 nm that are made in the endosomal part of a cell. They are involved in the intercellular regulation of pathophysiologic processes and serve as intercellular messengers that transport a variety of substances in a phospholipid bilayer membrane [4]. Exosomal circRNAs (exo-circRNAs) refer to the circRNAs discovered in exosomes [5]. When exosomes are released from cells, they are taken up by distant cells. Exosomes containing circRNAs regulate the TME to promote tumor cell proliferation, invasion, and metastasis [6, 7].

CircRNAs are closed, single-stranded RNA molecules without poly (A) tails and 5′-3′ ends, and compared to linear transcripts, they are more stable as they resist exonuclease-mediated destruction [8]. In 1979, endogenous circRNAs were discovered to be a byproduct of eukaryotic RNA splicing [9]. In 1986, the hepatitis delta virus caused circRNAs to be found in humans [10]. Almost 10,000 circRNAs have been identified, occurring naturally in many different organisms, from fungi to plants to vertebrates [11]. Currently, circRNAs are categorized into four classes: intergenic circRNAs, ecircRNAs, EIciRNAs, and exon–intron circRNAs [12]. Several studies have indicated that circRNAs are associated with various human disorders, including malignancies [13–17]. However, the mechanism and function of circRNAs have not been completely understood.

Exosomes are vesicles released from cancer cells; they carry circRNAs, which play an important role in cancer progression at multiple stages, including the proliferation of malignant tumors, formation of premetastatic niches, and metastasis of cancer cells to distant places [18, 19]. Li and his colleagues published the first study to know the expression levels of circRNAs in extracellular vesicles using the RNA-seq technique. They found that circRNAs are abundant at least twofold in exosomes than in cells and more stable [20]. In humans, around 60% of genes can express circRNA [21]. However, the tissue expression of these genes is still low, making up just 5–10% of the average mRNA expression in a specific tissue [22, 23].

Nevertheless, the relationship between exo-circRNAs and the promotion or inhibition of LC is still not well understood. Hence, this study provides recent studies on the functions and mechanisms of exo-circRNAs in LC and explains the connections between the dysregulation of exo-circRNAs and lung cancer progression. We also focused on possible therapeutic targets based on circRNA modulation and their potential function in promoting or inhibiting LC progression.

## Biogenesis of exosomes

**Fig. 1:**
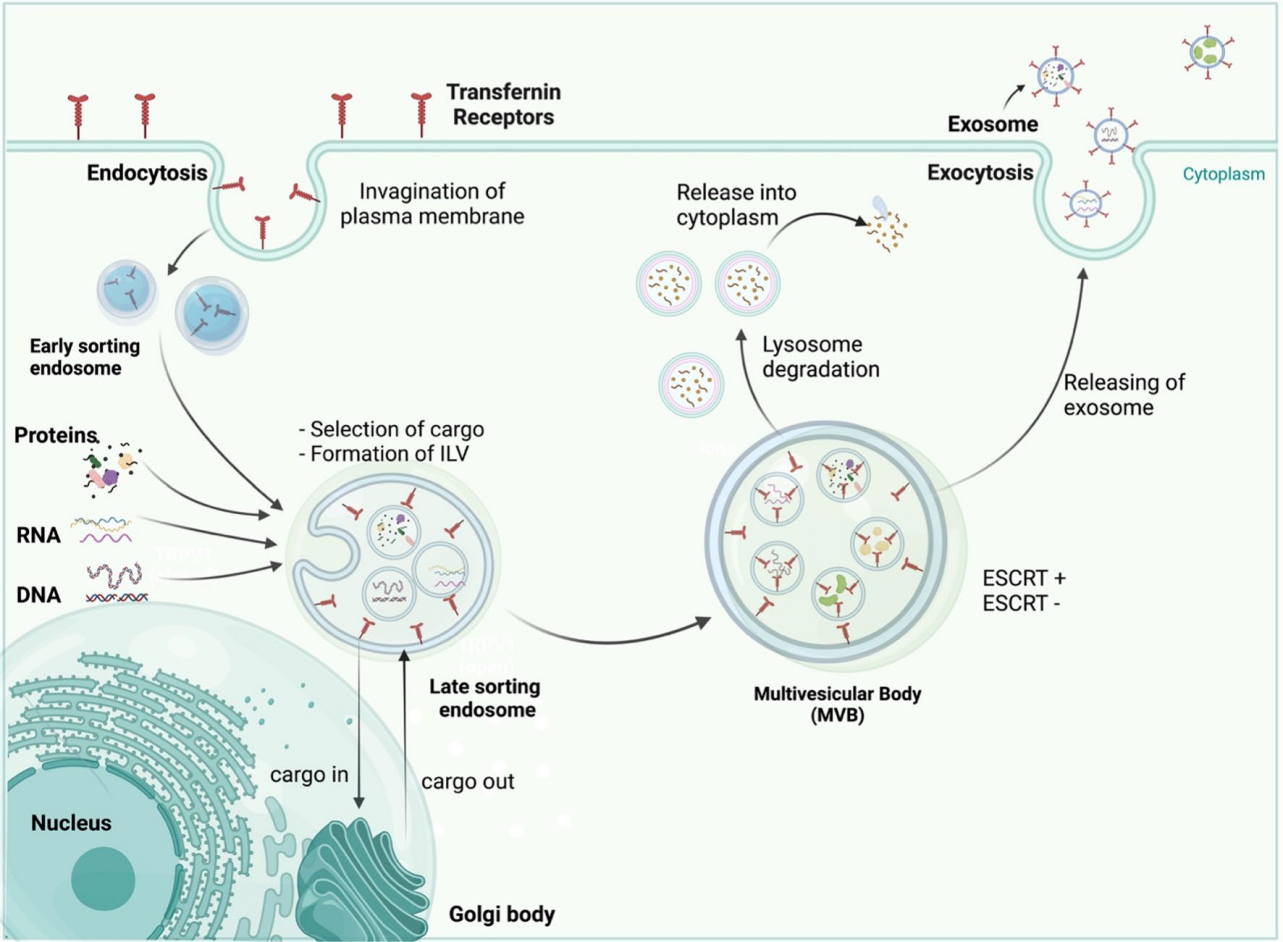
This illustration shows how exosomes are formed in the body and then released. Three processes contribute to exosome secretion: exosome biosynthesis, MVB transport to the cell membrane, and MVB fusion with the cell membrane

Exosomes originate from late endosomes, formed by the inward budding of the limited multivesicular body (MVB) membrane. The invagination of late endosomal membranes leads to the release of intraluminal vesicles (ILVs) inside massive MVBs [24]. Several proteins are taken to the invaginating membrane during this process. Meanwhile, the cytosolic components are taken up by the ILVs. Following fusion with the plasma membrane, most ILVs are discharged into the extracellular space, called exosomes, and move into body fluids [25, 26]. Eventually, these elements are taken by lysosomes, where they are broken down or released into the extracellular space after fusion with the plasma membrane [27] (Fig. 1). Endosomal-sorting complex that is required for transport (ESCRT) is necessary for both exosome biosynthesis and secretion [28]. Proteins such as ALIX, Tsg101, VPS4, and the four subunits of ESCRT (ESCRT-0, ESCRT-I, ESCRT-II, and ESCRT-III), make up ESCRT. ESCRT-0 carries out the sorting of cargo proteins into the lipid domain. Membrane deformation is carried out by the other ESCRTs I and II; the VPS4 complex is recruited to ESCRT-III, responsible for the vesicle neck scission and the dissociation or recycling of the ESCRT-III complex [29–31]. Through its interaction with the syndecan receptor, the exosomal protein Alix has been demonstrated to play a role in endosomal membrane budding and abscission and the selection of specific exosomal cargo [32]. In light of these findings, it was hypothesized that the ESCRT has a vital role in exosomal biogenesis.

After exosomes are released, they can send signals to target cells through endocytosis, a fusion of membranes, and interactions between receptors and ligands. Clathrin, caveolin, and lipid raft-mediated endocytosis can engulf exosomes into specific cells [33]. Endocytosed exosomes can either combine with nearby endosomes or be transported to lysosomes, where they are degraded [34]. The exosomal membrane also can bind to particular receptors on the plasma membrane of the recipient cell to initiate signaling pathways or to fuse with the plasma membrane of the recipient cell to distribute its contents [35–37].

## Biogenesis of circRNAs

**Fig. 2:**
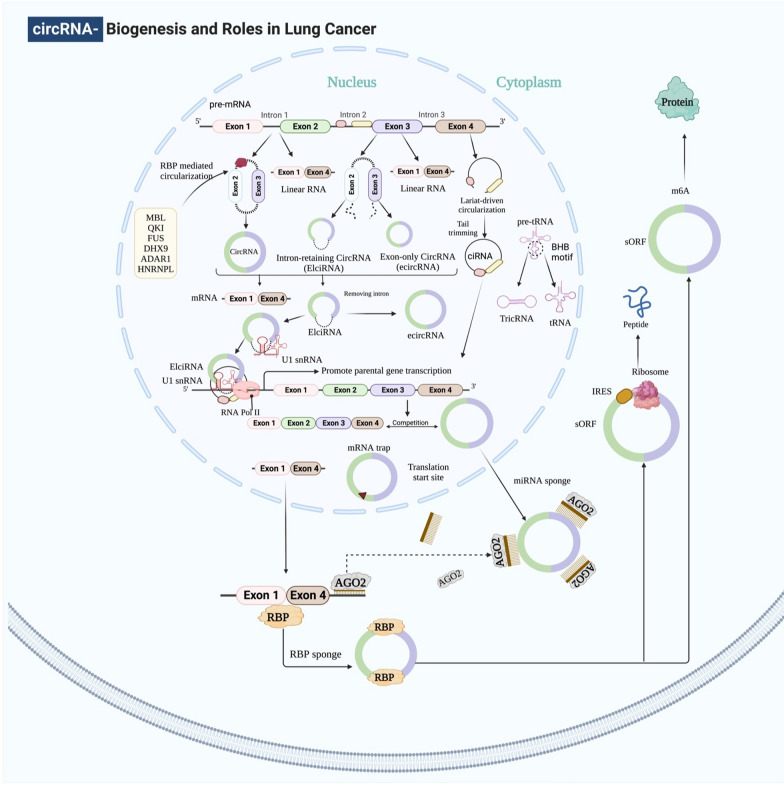
The process of biogenesis that occurs during lung cancer, in addition to the roles that exosomal circRNAs play in the disease

Synthesis of circRNAs from segments of pre-messenger RNAs can occur by back-splicing, a process in which the 5' splice donor joins with the 3' splice receiver through a phosphodiester bond. This biological process can create a circular structure with one or more exonic/intronic regions [38]. Numerous nuclear back-splicing and linear splicing processes have been described, including exon skipping, intron pairing, and RNA-binding proteins (RBPs) [39] (Fig. 2). The first is an RBP-assisted circularization process that generally involves the association of two neighboring exons and skipping the intronic region, producing an exonic-circRNA. Numerous RBPs regulate this process, including RNA helicase DHX9 [40], FUS [41], ADAR1 [42], NF90/NF110 [43], MBL [44], QKI [45], and heterogeneous nuclear ribonucleoprotein L [46].

Exon–intron circRNAs are made when two or more exons and their correlating introns circle. Intron pairing back-splicing is a popular approach in the conserved RNAs with many Alu repetitions in the sequences on either side. These Alu components work well together, promoting the configuration of hairpins and more back-splicing, leading to mono-EcircRNAs [47]. Another type of this category is the intronic circRNAs, but it is still unknown how these molecules are produced.

CircRNAs are exported into the cytoplasm after being synthesized in the nucleus. According to recent studies, the UAP56/URH49 helicases are actively involved in this size-mediated mechanism. Transferring molecules larger than 1300 nucleotides requires UAP55, whereas URH49 only interferes with short transcript exports [48]. Following their entry into the cytoplasm, circRNAs accumulate and regulate transcription by sponging certain types of miRNAs, as seems to be usual for most cells. Although the process by which circRNA degrades is still unknown, recent research has provided insights into this issue and shown some exciting pathways that explain circRNA disintegration. For example, Hansen et al. revealed a mechanism whereby Ago2 and miR-671 degrade circRNA-CDR1as [49]. Likewise, Park and his colleagues showed that a circRNA cleavage process is mediated by RNase P/MRP and outlined in N6-methyladenosine (m6A)-enriched circRNAs [50]. In recent work, Liu et al. [51] showed that certain circRNAs tend to form complicated duplexes, which renders them vulnerable to destruction by RNase L during viral infection.

### Biological functions of circRNAs

**Fig. 3:**
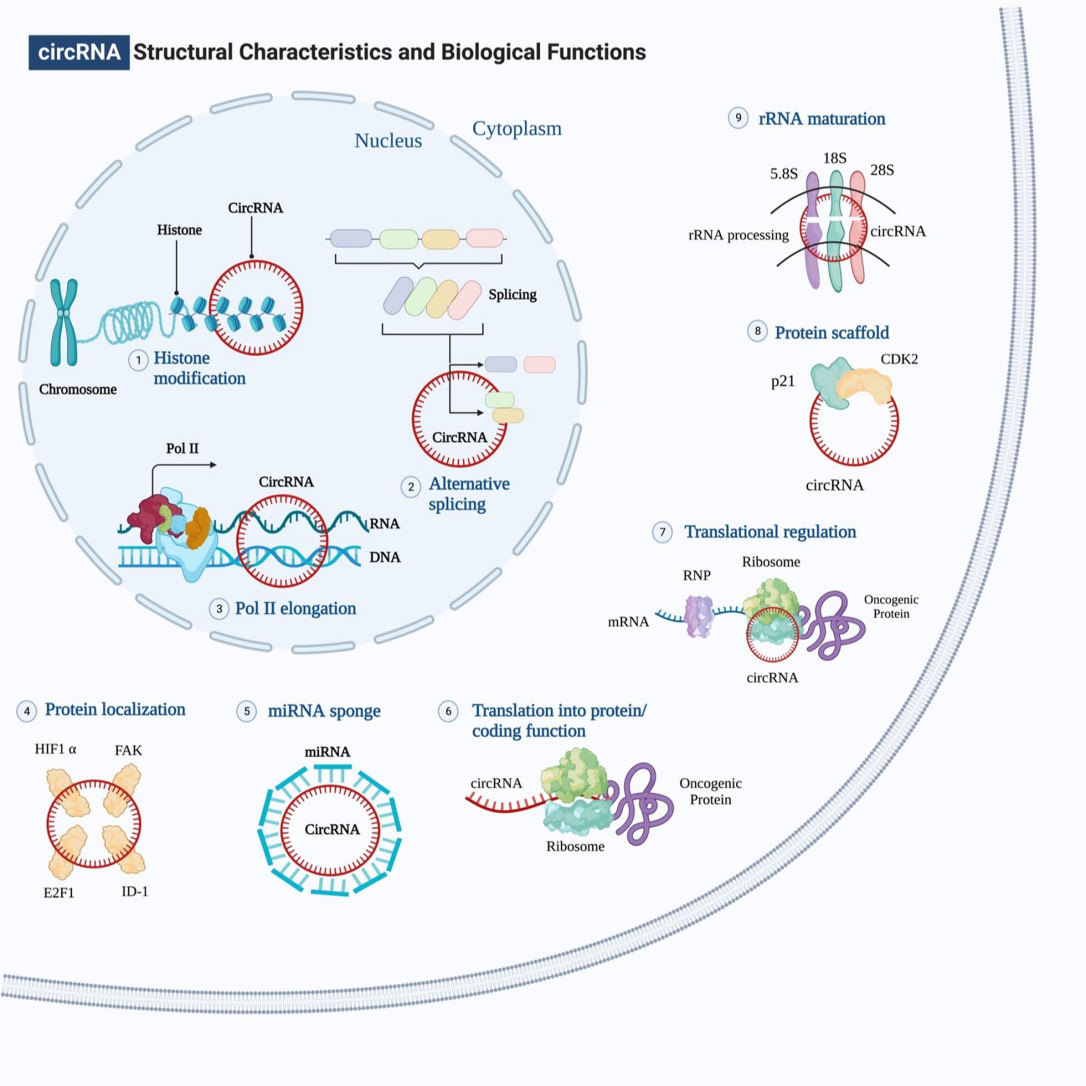
Structural characteristics and biological functions of circRNAs. Inside the cell, circular RNAs have many multiple roles to do. In the nucleus, circRNAs can silence a specific locus by interacting with the histone methylation pattern. They can also control the transcription of their own gene by interacting with RNA polymerase II. Finally, alternative splicing can be blocked by their competition with mRNA for splice sites. CircRNAs are found in the cytoplasm, where they can function as miRNA sponges, decoy certain transcription factors, be translated into proteins, bind with RNA-binding proteins to regulate translation of particular mRNAs, and serve as protein scaffolds. In addition to promoting cell death by interfering with the processing of pre-rRNA components, circRNAs can bind to some proteins and inhibit their signal transduction activity

Many studies have highlighted that circRNAs may control gene expression either directly or indirectly by binding to miRNAs, RBPs, and other regulators of gene expression and managing various biological processes (Fig. 3). The mechanisms of circRNAs that are used in regulating gene expression are as follows.

#### As miRNA sponge

The most critical function of circRNAs is to act as a miRNA sponge to regulate the expression of a target gene by inhibiting the activity of miRNA [52]. A single circRNA can bind to one or more miRNAs at one or more locations by perfect or near-perfect binding [53]. ‘‘Super sponges’’ like circRNAs are selectively attracted to miRNAs rather than other ceRNAs, such as lncRNAs and pseudogenes. The first example of a circRNA that functions as a miRNA sponge is CDR1as [54]. It has 74 miR-7 binding sites and is closely attached to AGO proteins. [55]. Gao and Ye et al. found that circ-SOX4 stimulated the growth of LUAD and activated the WNT axis by sponging miRNA-1270 and altering PLAGL2, providing a relevant conceptual framework for studying the therapeutic LUAD targets [56]. Additionally, circHIPK3 is derived from Exon2 of the HIPK3 gene, a key player in cell proliferation in human cancer, by sponging nine miRNAs with 18 binding sites into cells [57].

Despite the above, according to Militello et al. [58], some types of circRNAs, such as (circ_0005939 and circ_0013647) are unable to act as miRNA sponges. Therefore, additional work is needed to determine how circRNAs, miRNAs, and mRNAs work together.

#### Alternative splicing and transcriptional regulation

One of the most prevalent methods of controlling gene expression is alternative splicing, which is essential for enhancing functional proteins' complexity. Recently, it’s been shown that some circRNAs are highly concentrated in the nucleus, where they could potentially inhibit transcription. For instance, circURI1 may influence alternative splicing to promote cancer development and metastasis [59]. Likewise, EIciRNAs are circRNAs that have introns and exons [60]. Therefore, EIciRNAs are found in the nucleus and act as transcriptional regulators [61]. Besides, EIciRNAs regulate RNA polymerase II (Pol II) activity and trigger the transcription of parental genes [62]. EIciRNAs and Pol II work together to promote transcriptional initiation by making it easier for Pol II to bind with the core promoter of EIciRNA parent genes [63]. Similarly, the EIciRNAs and the U1 snRNA (small nuclear ribonucleoprotein) attach in an RNA-RNA manner, which makes it possible for the EIciRNAs and pol II to interact with one another [64]. Additionally, circRNAs, such as exon–intron circular RNAs (circPAIP2, circEIF3J), could attach to Pol II and control their host gene expression [65]. Accordingly, these studies suggest that intron-derived circRNAs are responsible for regulating the transcription process in the nucleus.

#### Translation

Endogenous circRNAs have been shown recently to be capable of protein translation. The protein-coding capacity of circRNAs was previously thought to be low, but it has been proven that circRNAs with IRES or N6-methyladenosine modifications can often be translated into peptides [66, 67]. In eukaryotic cells, untranslated regions (UTR) are necessary for the beginning of the translation process, specifically 5' and 3' positions. Due to the absence of 5' and 3' ends, circRNAs were previously categorized as ncRNAs. Growing data proved that circRNAs might be able to code for proteins since they can be coupled to polysomes, and some of them have AUG start codon in addition to putative ORFs with favorable lengths [68, 69]. According to the Legnini et al. study, the back splicing result of ZNF609 exon 2, known as circ-ZNF609, can be translated into a protein in both a splicing-dependent and a splicing-independent manner throughout the process of myogenesis [70]. However, it's not clear how standard circRNA translation occurs yet, and it's also not clear what the translated proteins might perform or what components are involved in the process. Despite its novelty and significance, the study of how circRNAs are translated into peptides or proteins has been published in only a few studies due to limitations in analysis and validation methodologies.

#### CircRNAs and RNA-binding proteins

Recent research has shown that circRNAs work like miRNA sponges, inhibiting miRNA function while also taking part in splicing target genes, translating genes into proteins, and interacting with RNA-binding proteins (RBPs). Interaction with RBPs is a crucial component in the actions of circRNAs, which include biogenesis, translation, control of target genes, and extracellular transport [71]. For instance, circBIRC6 is highly represented in the Ago2 binding complex and mediates pluripotency in hESCs by inhibiting differentiation through direct interactions with miR34a and miR145 [72]. Similarly, stat3 binding circAmotl1 and increasing nuclear translocation enhanced cell activity. Nuclear Stat3 would bind to Dnmt3a's promoter, increasing transcription and translation. Then, the miR-17 promoter is demethylated by Dnmt3a, which reduces the production of miR-17-5p [73]. These show a feedback loop in which circRNA-based RBPs bind together and perform different regulatory functions.

## Implication of exosomal circRNA in lung cancer progression

According to several studies, exosomes contain a variety of non-coding RNAs (ncRNAs), including miRNA, lncRNA, circRNA, and rRNA [74–76]. In contrast to cells that release circRNA, also circRNAs are highly concentrated and persistent in exosomes, particularly in those generated from tumors.

Exosomal circRNAs are involved in several critical biological processes that promote or inhibit cancer [77, 78]. More evidence suggests that exo-circRNAs play a crucial role in several malignancies, including lung cancer, through different mechanisms (Table 1). Exosomal circRNAs have a similar physiological role in malignancies via the miRNA sponge [79]. For instance, circ 0013958, a molecular sponge for miR-134 in LC, was connected with lymphatic metastasis and the TNM stage [80]. Likewise, circFARSA promotes the progression of LC through sponging miR-326 and miR-330-5p, thereby allowing these miRNAs to lose their control of the FASN oncogene, which is the gene that causes cancer [65, 81]. Moreover, exosomes containing exo-hsa_circRNA_0056616 were highly expressed in tissues from lung adenocarcinomas that had lymph node metastases [82]. Similarly, overexpression of circCCDC66 by STAT3 increases the growth of NSCLC by affecting the miR-33a-5p/KPNA4 pathway [83]. Furthermore, circABCB10 altered the miR-584-5p/E2F5 axis to accelerate the development of NSCLC [84]. On the other hand, exosomal circPVT1, which is produced by LC cells, activates the axis of miR-124-3p/EZH2 to polarize macrophages and increase lung tumor cell invasion and migration [85]. Exo-circRNAs, taken as a whole, might be an important factor in the advancement of LC. Table 1 lists the patterns of oncogenic exo-circRNA expression, along with the genes they target and the mechanisms of actions with their functions.

**Table 1:** Oncogenic exo-circRNAs and their role in lung cancer

Exo-circRNA	Number of clinical samples	Types of samples	Animal model	Expression	Target genes	Mechanisms	Functions	Refs
CDR1-AS	104 LUAD patients	PAEC, LUAD PC9, A549	–	Up	PTX, CDDP	EGFR/PI3K pathway	Independent prognostic biomarker for LUAD patients	[165]
hsa_circ_0014235	Tumor tissues 35 samples and adjacent 35 samples	A549, H1299, 16HBE	Nude mice	Up	miR-520a-5p, DDP, CDK4	miR-520a-5p/CDK4 regulatory axis	An increase in DDP resistance and promotion of cancerous cell activity	[100]
hsa_circ_0056616	42 lung adenocarcinomas with lymph node metastases, 48 without	PC9, PC14	–	Up	CXCR4	–	CXCR4 knockdown inhibits colony formation, cell proliferation, migration, and invasiveness	[82]
Circ-MAN2B2	–	BESA-2B, A549, H226, H1299, H446	–	Up	miR-1275, FOXK1	CircMAN2B2/miR-1275/FOXK1 signaling	Act as an oncogene, which promotes lung cancer cell proliferation and invasion	[166]
hsa_circ_0013958	49 pairs of LAC samples	A549, H1299, BEAS-2B	–	Up	miR-34, CCND1	–	Encouraging cell growth and invasion while discouraging cell death	[167]
hsa-circRNA-002178	105 paired LUAD and noncancerous tissue samples	95D, PC9, A549, BEAS-2B	–	Up	miR-34a, miR-28-5p, PDL1, PD1	–	Increase PDL1 and PD1 expression in tumor cells	[168]
Circ-CPA4	NSCLC patients (N = 50)	cA549, H1299, SK-MES-1, Calu-3, HBE	Nude mice	Up	miR-134, let-7 miRNA, PD-L1	Let-7 miRNA/PDL-1 axis	Immunity evasion	[169]
CircFARSA	10 pairs of tumor and adjacent normal tissues	A549	–	Up	miR-330-5p, miR-1270, miR-1178-3p, miR-620, miR-326	–	A novel biomarker for NSCLC	[170]
Circ_0014130(circPIP5K1A)	–	H1299, PC9, H1975, A549, H1650, BEAS‐2B	Nude mice	Up	miR-600, HIF‐1α gene 3′‐UTR	CircPIP5K1A/miR‐600/HIF‐1α axis	MiR600 reduced HIF1-mediated metastasis and cancer growth	[171]
Circ_RAD23B	40 NSCLC samples and paired adjacent normal tissue specimens	H1299, H1581, H358, A549, 16HBE	–	Up	miR593-3p, miR-653-5p, CCND2, TIAM1	MiR-593e3p/CCND2 axis, and miR-653e5p/TIAM1 pathway	Function as oncogene by miRNA sponge	[172]
CircPVT1	–	–	–	Up	miR-124-3p, EZH2	MiR-124-3p/EZH2 axis	Exosomal circPVT1 enhances proliferation and metastasis by polarizing macrophages through miR-124-3p/EZH2	[85]
Circ-MEMO1	Tissue samples 52 tumor and adjacent normal	H1650, A549, H1299, PC9, HBE	Nude mice	Up	miR-101-3p, 3′’ UTR of KRAS	MiR-101-3p/KRAS Axis	MiR-101-3p targeting KRAS increased NSCLC progression and glycolysis	[173]
	Serum samples; 30 patients and 25 healthy							
Circ-PRMT5	90 pairs of cancer and adjacent normal tissues	A549, 95-D, HCC827, H1299, SK-MES-1, HBE	Nude mice	Up	miR-377/382/498	MiR-377/382/498-EZH2	Circ-PRMT5 promotes NSCLC growth by miR-377/382/498 sponging and upregulating EZH2	[174]
						pathway		
CircHIPK3	3 different primary lung cancer	A549, BEAS-2B	–	Up	miR124	CircHIPK3-miR-124 pathway	Promotes lung cancer cell progression via miRNA sponging	[175]
	patients							
CircRNA CCDC66	628 patients with newly diagnosed NSCLC	H125, H23, H226, H838, H1437, H2009, H2087, A549, H125, H23, H838, H1437, H2009, H2087, A549	–	Up	ATAD3A, SAE2, CCDC66, EGFR	HGF/c-Met axis	Enhance LADC cell EMT and drug resistance	[176]
Circ-STXBP5L		–		Up	miR-224-3p and miR-512-3p	–	Circ-STXBP5L target miRNAs, causing LC progression	[177]
hsa_circ_0000064	–	A549, H1299	–	Up	Caspase-3, 9, BAX, p21, cyclin D1, CDK6, MMP-2, 9	–	Induces cancer cell proliferation, apoptosis and metastasis	[178]
Circ-FOXM1	80 NSCLC patients	H1299, A549, SK-MES-1, Calu-3HBE	–	Up	miR-1304-5p, PPDPF, MACC1	Circ-FOXM1/miR-1304-5p/PPDPF/MACC1 axis	Increases cellular growth and proliferation by sponging miR-1304-5p to target PPDPF and MACC1	[179]
Circ_0047921	patients (n = 60)	H1299, A549, H1650, Calu3, SK-MES1, BEAS-2B	Nude mice	Up	miR-1287-5p, LARP1	Circ_0047921/miR-1287-5p/LARP1 axis	Circ0047,921 serves as miR-1287-sponge, controlling LC cell proliferation, migration, and glycolysis	[180]
Circ-0006006	–	A549, H1299	Nude mice	–	miR-924, SRSF7	MiR-924/SRSF7 axis	Accelerated NSCLC development by regulation of SRSF7 expression via miR-924 sponging	[181]
Circ_0008717	48 NSCLC patients and 48 control samples	A549, H1299, BEAS-2B	Nude mice	High expression	miR-1287-5p, PAK2	miR-1287-5p/P21-mediated kinase 2 (PAK2) pathway	Promotes carcinogenesis in NSCLC by increasing expression of PAK2 via miR-1287-5p sponging	[182]
CircMAGI3	30 NSCLC patients	H322, H460, A549, H1299, NHBE	Nude mice	up	HDGF, miR-515-5p	CircMAGI3/miR-515-5p/HDGF pathway	Stimulates cell glycolysis and NSCLC cell proliferation	[183]
Circ-ABCB10	40 NSCLC patient samples	SPC-A1, HCC827, H1975, H1650, PC9, A549	Nude mice	up	MiR-584-5p, E2F5	MiR-584-5p/ E2F5 pathway	Participate in the upregulation of E2F5 expression by sponging miR-584-5p	[84]
hsa_circ_0062389	33 paired of NSCLC samples	H1650, H23, H522, A549, H1703, H460, BEAS-2B	–	up	MiR-103a-3p, CCNE1	MiR-103a-3p/CCNE1 axis	Utilize miR-103a-3p as a sponge to control CCNE1 expression in LC	[184]
Circ_0072088	20 patients with LUAD	H1299, H1975, H520, H827	–	Up	MiR-1261, PIK3CA	Circ_0072088/miR-1261/PIK3CA regulatory pathway	Tumorigenesis and progression of LUAD	[185]
Circ_0007385	–	–	Nude mice	Up	MiR-1253, FAM83A	MiR-1253/FAM83A axis	Promoted NSCLC cell proliferation and stemness	[186]
CircTUBA1C	30 pairs of LC tissue samples	Calu-3, A549	Nude mice	Up	MiR-143-3p, Cyclin B1, PCNA, BAX, caspase-3	CircTUBA1C/miR-143-3p axis	CircTUBA1C sponges miR-143-3p to promote NSCLC	[187]
Circ-PITX1	40 patients with primary NSCLC	H1975, A549, BEAS	Nude mice	Up	MiR-30e-5p, ITGA6	MiR-30e-5p/ITGA6 axis and ITGA6/PI3K/Akt pathway	MiRNA sponge	[188]
CircFECR1	35 moderate and 26 extensive SCLC patients	NCI-H460,NCI-H446, NCI-H2170,NCI-H1688, NCI-H1299, HCC-827	Nude mice	Up	MiR584-3p, ROCK1	MiR584–ROCK1 pathway	miR584-3p ensnared and deactivated by FECRs, which triggered the ROCK1 pathway	(189)
CircRNA-102481	58 NSCLC patients	PC9	–	Up	MiR-30a-5p sponge, ROR1	CircRNA_1024810/miR-30a-5p/ROR1 axis	promotes EGFR-TKI resistance through the miR-30a-5p/ROR1 pathway	[190]
CircSATB2	59 NSCLC and normal tissue samples	BEAS-2B, A549, H460, H1299, H226, MES-1	–	Up	MiR-326, FSCN1	–	Encourages LC to grow, spread, and invade	[191]
Hsa_circ_0002130	28 osimertinib-resistant LC (non-response) and 32 sensitive (response)	HCC827, H1975	Nude mice	Highly expressed	MiR-498, GLUT1, HK2, LDHA	–	Osimertinib-resistant NSCLC promotion	[192]
Circ_100876	–	A549, NCI-H23	–	Up	Targeting miR-636, RET	MiR-636/RET axis	CircRNA 100876 downregulation decreased NSCLC via the miR-636/RET pathway	[193]
Circ_0002346	45 NSCLC tissue specimens	HBE, A549, H1299	Nude mice	Up	miR-582-3p, STXBP6	miR-582-3p/STXBP6 pathway	Circ 0002346 sponges miR-582-3p to promote STXBP6 in NSCLC cells	[194]
Hsa_circ_0018818	30 pairs of LC and normal tissues	A549, NCI-H1650, PC-9, 293 T, NCI-H441, BEAS-2B	Nude mice	Up	miR-767-3p, NID1	miR-767-3p/NID1 signaling pathway	Targeted shRNA decreased NSCLC cell growth, invasion and induced the apoptosis process	[195]

### Exosomal circRNAs and EMT

**Fig. 4:**
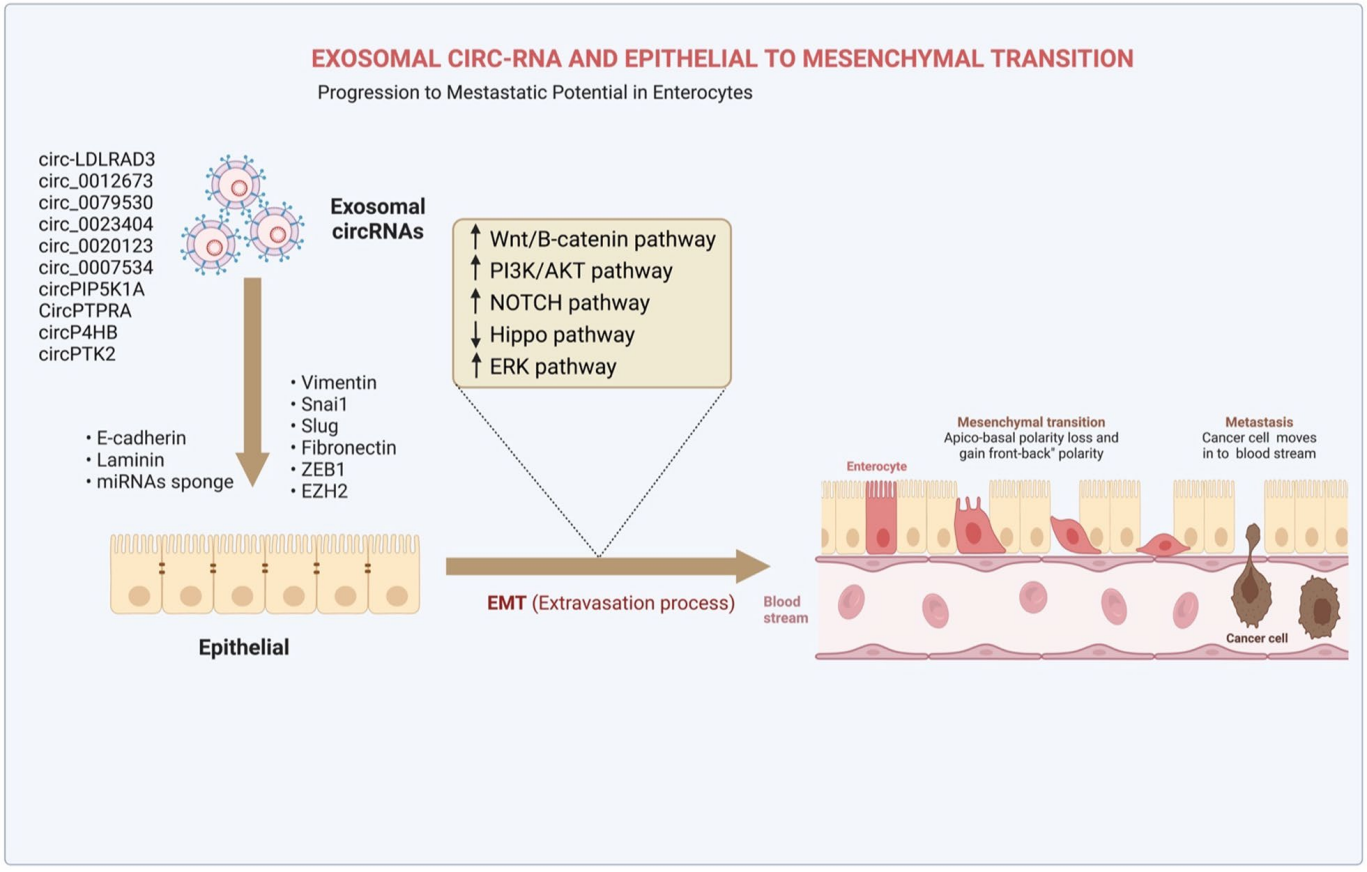
This illustration highlights the key roles of exosomal circRNAs in the EMT process in lung cancer. Exosomal circRNAs which are overexpressed and play oncogenic functions by promoting EMT processes through promoting or/and inhibiting different pathways in lung cancer

Once epithelial cells gain motility, a process known as the epithelial-mesenchymal transition (EMT) takes place and adopts a mesenchymal phenotype while retaining their invasive abilities [86]. Such an approach has been extensively seen in various biological phenomena, such as embryogenesis, fibrosis, cancer growth, and metastasis [87]. Like other malignant tumors, LC can spread and invade tissue due to the EMT process [88]. A high abundance of circRNAs is observed in LC, and some of them play oncogenic functions by promoting EMT processes in vitro (Fig. 4). For example, Inhibition of microRNA-137 by circ-LDLRAD3 led to an increase in glutamine transporter, a member of the SLC1A5in NSCLC cells, hence promoting proliferation and EMT [89]. Specifically, SLC1A5 was crucial for developing and controlling LC, and its inactivation was found to reduce the viability of LC cells [90]. Additionally, circ 0012673 enhances the proliferation and invasion of LUADs [91]. Reducing circ 0012673 levels inhibited cell growth, motility, and EMT via upregulation of LIM domain kinase 1in LUAD cell lines while simultaneously triggering apoptosis via miR-320a targeting [91]. According to Li et al., overexpression of hsa circ 0079530 stimulated cancer cells to migrate and invade through controlling EMT processes [92]. Similarly, EMT-related protein expression is regulated by hsa circ 0023404 through modulation of the miR-217/zinc finger E-box-binding homeobox 1 (ZEB1) axis and promoting LC cell growth [93].

**Fig. 5:**
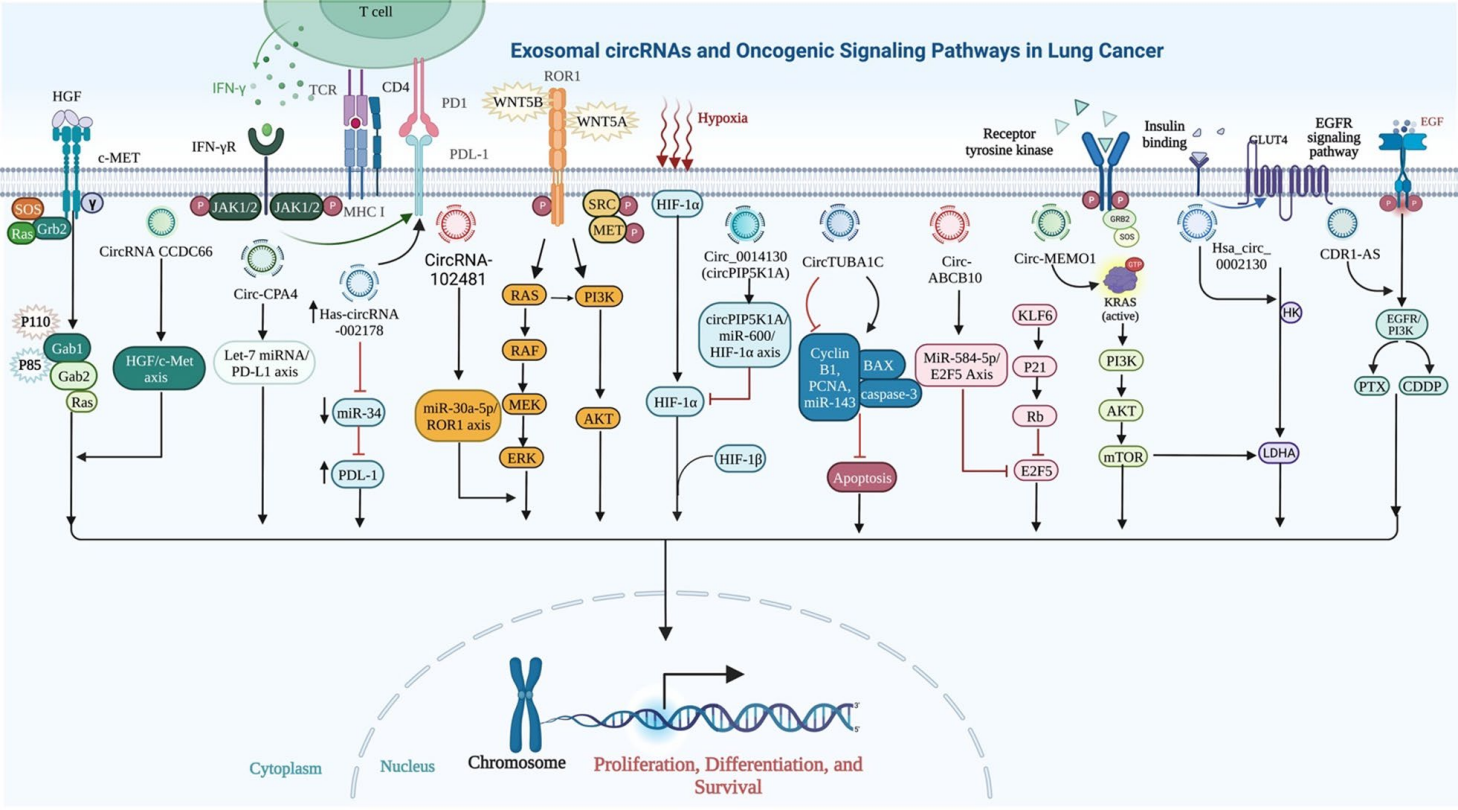
Illustration shows the connection between oncogenic signaling pathways and exosomal circRNAs in LC. CircRNA can promote tumor cell proliferation, invasion, migration, and survival by targeting particular genes and sponging various types of microRNAs, such as miR-101-3p, miR-498, miR-584-5p, miR-143-3p, and miR-600. CircRNA can act as an oncogene and promote the proliferation of cancer cells by involving in several essential signaling pathways in lung cancer, including EGFR/PI3K, miR-101-3p/KRAS, and miR-584-5p/E2F5 pathways

Furthermore, EMT plays a crucial role in LC, and numerous in vivo and in vitro studies have demonstrated that oncogenic circRNAs speed up this process through a number of pathways (Fig. 5). For instance, Qu and colleagues revealed that hsa circ 0020123 inhibited LC apoptosis by decreasing miRNA-144 and increasing ZEB1 and EZH2 expression [94]. Their results demonstrated that knocking down hsa_circ_0020123 slowed the growth and spread of LC cells. According to a recent study, circPIP5K1A functioned as a miR-600 sponge to increase LC development by increasing HIF-1α and inhibiting miR-600's effect on EMT-related proteins [95]. Similarly, in vitro experiments showed that circP4HB stimulated EMT processes in LC via sponging miR-133a-5p, as demonstrated by an increase in vimentin expression [96].

**Fig. 6:**
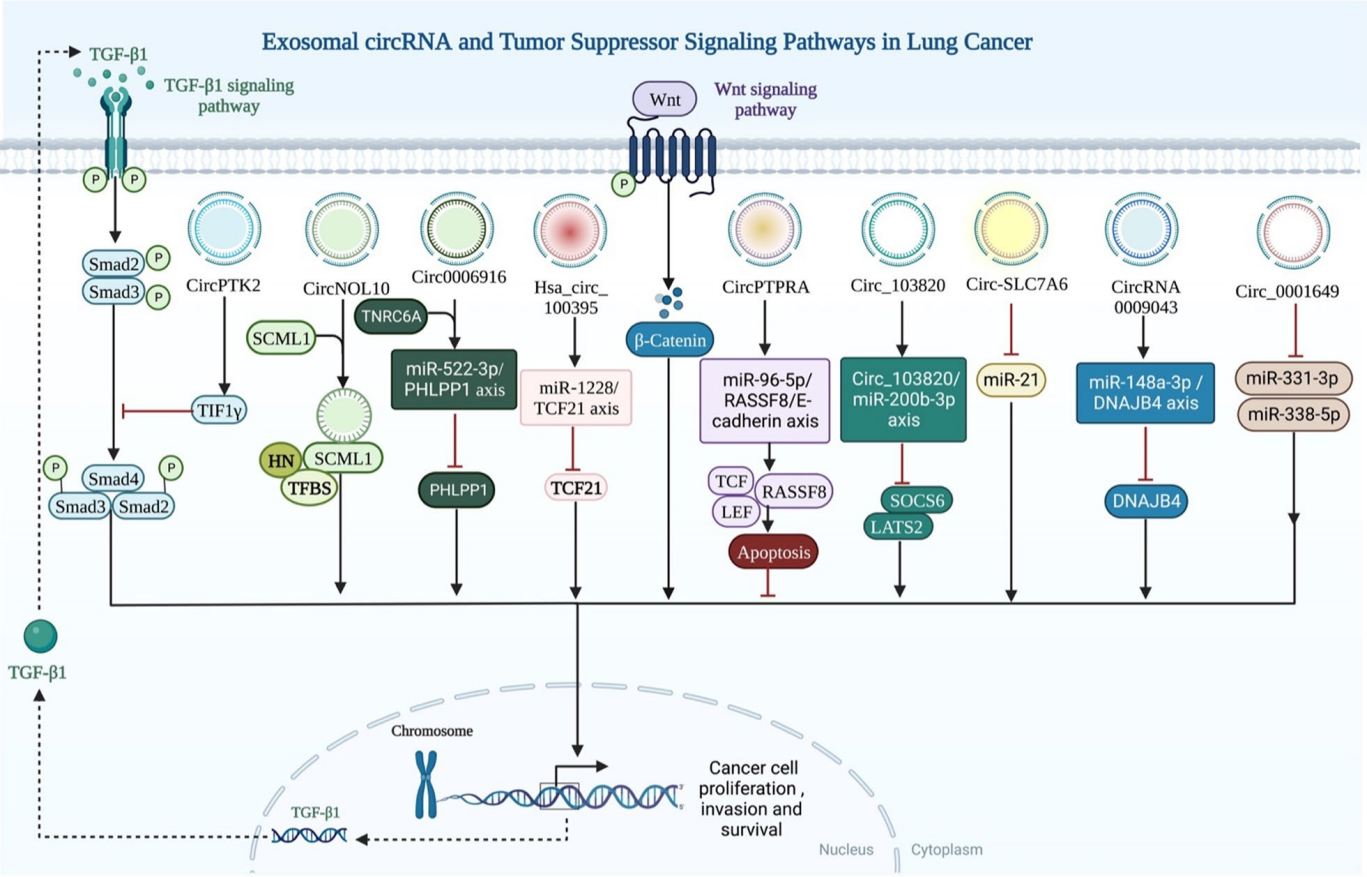
Illustration shows the relationship between tumor suppressor signaling pathways and exosomal circRNAs in lung cancer

Despite this, a number of circRNAs are significantly suppressed in vitro and in vivo in LC, and, through positively regulating the EMT process, they prevent cancer progression (Table 2). For example, circPTK2, a miRNA sponge, was positively correlated with TIF1-y expression in human NSCLC tissue. [97]. Furthermore, overexpression of circPTK2 was found to elevate TIF1-y levels and suppress the TGF-β signaling pathway (Fig. 6). Additionally, by entrapping miR-96-5p and increasing the expression of RASSF8, circPTPRA inhibited EMT processes in LC cells and decreased cancer cell metastasis in a mouse xenograft model [98]. These results have given new insights into the EMT-mediated perspectives of the function of circRNAs within LC.

**Table 2:** Exo-circRNAs which are functioned as tumor suppressors in lung cancer

Circ-RNA	Number of clinical samples	Types of specimens	Animal model	Expression	Target Genes	Mechanisms	Functions	Refs
CircNOL10	61 pairs of cancerous and paracancerous lung tissue samples	A549, H1299, H226, H460, H661, SK-MES-1, BEAS-2B	Nude mice	Down	ESRP1, SCML, HN	–	Promotes SCLM1-mediated transcriptional regulation, hence suppressing LC development	[196]
Circ0006916	49 patients	16HBE-T, A549, H460, 16HBE	–	Down	miR-522-3p, TNRC6A, PHLPP1	–	Upregulating PHLPP1 with miR-522-3p inhibits cellular proliferation and tumor growth	[197]
hsa_circ_100395	69 pairs of LC tissues and normal tissues	A549, H460	Nude mice	Down	miR-1228, TCF21	miR‐1228/TCF21 axis	miRNA sponge	[198]
CircPTK2	73 pairs of LC tissues and normal tissues	A549,H1299, H1650, SPC-A1, Calu3, H226, H520, SK-MES-1, BEAS-2B	Nude mice	Down	miR-429/miR-200b-3p, 3’-UTR of TIF1γ, TGF-β	–	Targeting TIF1 to reduce TGF- γ induced EMT as miR-429/miR-200b-3p sponges	[199]
Circ_0001649	53 paired of tissue specimens	A549, H358, H1299, H1581, 16HBE	Nude mice	Down	miR-331-3p and miR-338-5p	Circ_0001649 miR-331-3p/miR-338-5p regulatory pathway	Represses LC development by sponging miR-331-3p and miR-338-5p	[200]
Circ_103820	20 paracarcinoma and lung cancer pairs	SPCA1, A549, HEK-293 T	–	Down	miRNA-200b-3p, LATS2, SOCS6	Circ_103820/miRNA-200b-3p axis	Lung cancer miR-200b-3p sponge modulates LATS2 and SOCS6 expression	[201]
Circ-SLC7A6	110 pairs of NSCLC and precancerous normal tissues	A549, H460	Nude mice	Down	miR-21	Circ-SLC7A6/miR-21 axis	circ-SLC7A6 suppressed LC proliferation through sponging miR-21	[202]
CircPTPRA	NSCLC patients (n = 34)	H522, H23, H1755, BEAS-2B	Nude mice	Down	miR-96-5p, RASSF8	miR-96-5p/RASSF8/E-cadherin pathway	prevents LC cells from undergoing EMT and spreading by sponging miR-96-5p	[203]

### Exosomal circRNAs and cell proliferation

**Fig. 7:**
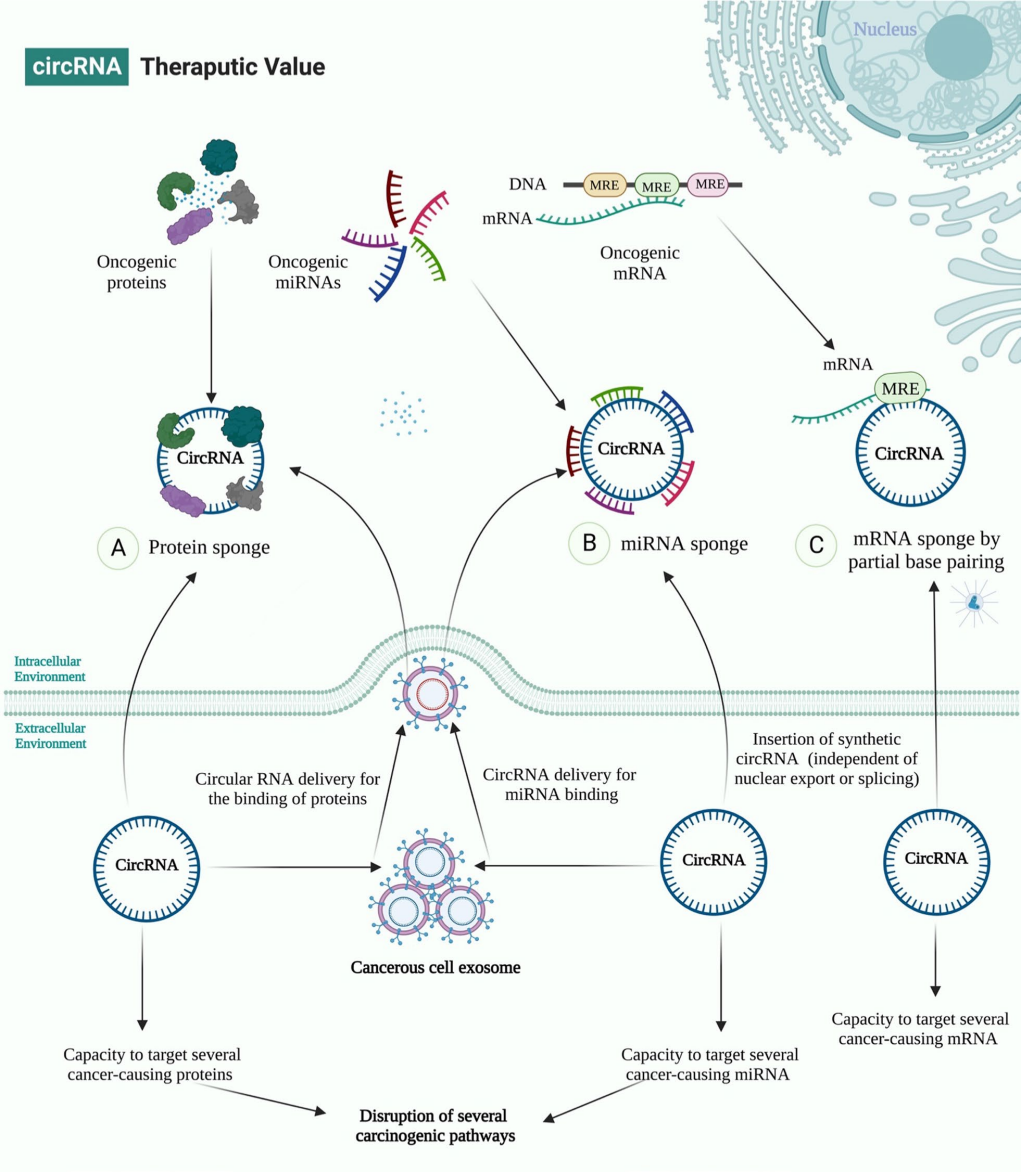
Circular RNAs have been demonstrated to have therapeutic promise and have a potential to be applied in the treatment of a wide range of diseases including lung cancer

Dysproliferation is a significant contributor to tumor progression, therefore the control of cell growth has attracted more attention [99]. Recently, exo-circRNAs have been shown to influence cell proliferation in a variety of malignancies, including lung cancer. Fig. 7 For instance, Xu et al. found that hsa_circ_0014235 promoted tumor development in non-small cell lung cancer through modulating the miR-520a-5p/CDK4 regulatory axis [100]. They revealed that hsa_circ_0014235 increased tumor growth by promoting cell proliferation, migration, and DDP resistance in vivo. In addition, Ying et al. demonstrated that the expression of circPVT1 was upregulated and stimulated cell proliferation in blood-derived exosomes isolated from lung cancer patients [85]. They found that exo-circPVT1 promotes LC proliferation through targeting the miR-124/EZH2 axis and induces macrophage polarization. Furthermore, circ-FOXM1 Table. 3 increases cell proliferation in NSCLC by targeting PPDPF and MACC1 with miR-1304-5p and is directly linked to lymph node invasion, a high TNM grade, and a poor prognosis [101]. Likewise, in NSCLC tissues and cells, Wei et al. proved that the levels of circ-FOXM1 and ATG5 were elevated, whereas the level of miR-149-5p was downregulated. Circ-FOXM1 knockdown reduced autophagy and cancer cell survival [102]. They observed that miR-149-5p functioned by inhibiting ATG5 expression, and circ-FOXM1 functioned by suppressing miR-149-5p expression. Similarly, exo-circaARHGAP10 expression level was increased in NSCLC tissues and serum samples. In vitro proliferation and glycolysis of NSCLC cells were suppressed by circARHGAP10 knockdown, while tumor growth was inhibited in vivo [103]. Recently, exosomes, according to Hongya et al., were responsible for transmitting circVMP1, which accelerated the proliferation of NSCLC and DDP resistance by targeting the miR-524-5p-METTL3/SOX2 axis [104].

**Table 3:** The potential role of exo-circRNAs as biomarkers in the diagnosis and treatment of lung cancer

CircRNA	Role/Function	Regulation	Mechanism	Sample	Refs
Circ_0047921	Biomarker	↑	–	Serum Exosome	[204]
Circ_005628	Biomarker	↑	Circ_005628/miR-1244/TRIM44	Serum Exosome	[205]
circ_0001492 circ_0001346 circ_0000690	Biomarker	↑	circ_0001492/miR-93-5p	Plasma Exosome	[206]
Circ_0001439	Biomarker	↑	–	Plasma Exosome	[207]
CircFARSA	Biomarker/ enhances NSCLC metastasis	↑	CircFARSA/PTEN/PI3K/AKT axis	Cell Line Exosome	[116]
hsa_circ_0069313	Biomarker	↑	–	Serum Exosome	[208]
Circ_0043278	Increased expression of ROCK1, CDKN1B, and AKT3 promotes proliferation, invasion, and migration	↑	miR-520f /ROCK1/CDKN1B/AKT3 axis	Cell Line Exosome	[209]
Circ CDYL	Sponges miR-185-5p and controls TNRC6A to suppress cell growth and trigger cell death	↓	Circ CDYL/miR-185-5p/TNRC6A axis	Cell Line Exosome	[210]
CircARHGAP10	Boost cell division, migration, invasion, and glucose metabolism	↑	CircARHGAP10/miR-638/FAM83F axis	Serum Exosome	[211]
hsa_circ_0002130	Involves facilitating resistance to osimertinib	↑	Hsa_circ_0002130/miR-498 axis	Serum Exosome	[192]
Circ_0008928	Upregulation of miR-488 and HK2 in CDDP-resistant LC promotes cell proliferation, migration, and glycolysis metabolism	↑	Circ_0008928/miR-488/HK2 axis	Serum Exosome	[212]
CircSETDB1	Promotes growth and metastasis	↑	CircSETDB1/miR-7/Sp1 axis	Cell Line Exosome	[213]
circRNA-002178	Immune escape	↑	CircRNA-002178/miR-34/PDL1	Plasma Exosome	[168]
Circ_0076305	DDP resistance in NSCLC is controlled by upregulating ABCC1 expression via miR-186-5p sponging	↑	Circ_0076305/miR-186-5p/ABCC1 axis	Cell Line Exosome	[214]
CircVMP1	miR-524-5p-METTL3/SOX2 axis targeting promotes NSCLC development and DDP resistance	↑	miR-524-5p-METTL3/SOX2 axis	Cell Line Exosome	[104]

In contrast to the above, several circRNAs act as tumor suppressors, and they inhibit lung cancer cell proliferation. For example, the expression level of circ_0006677 was lower in LC cells and NSCLC tissues from patients compared to nearby healthy tissues. Poorer patient survival was considerably related to lower expression of circ 0006677 [105]. Circ_0006677’s overexpression drastically reduced NSCLC cells' capacity for proliferating, invading, and metabolizing glucose. By controlling the expression of the signal transducer inhibitor SOSC2 through sponging miR-578, circ_0006677 could prevent the growth of NSCLC and glycolysis [105]. Additionally, Shi et al. found that hsa_circ_0069244 also acts as a sponge for miR-346 to limit the proliferation of lung cancer via regulating XPC expression [106]. Recently, in both NSCLC tissues and cell lines, hsa_circ_0003176 had the typical characteristics of circRNAs, which were downregulated. Functionally, hsa_circ_0003176 was overexpressed, which prevented NSCLC cells from proliferating, invading other cells, and growing both in vitro and in vivo [107]. These findings might improve our understanding of the molecular processes behind the development of NSCLC into a malignant state.

### Exosomal circRNAs mediated regulation of angiogenesis

Tumors are distinguished by their capacity for unrestricted reproduction, independent maintenance of their nutritional status, and aberrant regulation of their cellular energy metabolism [108]. Angiogenesis is essential to the microenvironment in which this severe and uncontrolled growth occurs [109]. When the tumor's "angiogenesis switch" is activated, the vascular system responds or becomes more dynamic and produces new blood vessels to supply the growing tumor [110]. Exo-circRNAs have recently been found to play a crucial role in tumor angiogenesis [111]. For example, Yang et al. showed that the abundance of circ_0006988 was increased in tissues and NCSLC cells. They proved that the angiogenesis process was slowed by silencing circ_0006988 [112]. Circ_0006988 can sponge miR-491-5p, which leads to overexpressing of MAP3K3. The growth of xenograft tumors was also inhibited when circ 0006988 was silenced or knocked down. This was accomplished by reducing tumor-promoting angiogenesis [112]. Moreover, the expression of circ_0016760 was significantly higher in NSCLC tissues and cells than in normal lung tissues. Because of its ability to behave as a miR-29b sponge, circ 0016760 was able to prevent miR-29b from binding to HIF1A. Furthermore, circ_0016760 silencing inhibited cell proliferation, invasion, and angiogenesis or tube formation [113].

There has only been a limited of research done on how circRNAs participate in the process of LC angiogenesis. However, circRNA-based molecular therapy may be an option for treating LC due to its advantages, such as its low molecular weight and high stability.

### Exosomal circRNAs and metastasis

Tumor metastasis is the term for the spread of malignant tumor cells from their initial site and metastasis is the main factor that leads to cancer-related mortality [114]. Adhesion, disintegration, and migration are the three main steps of tumor cell metastatic progression. Through miRNA sponging, circRNAs regulate NSCLC invasion and metastasis. For instance, the serum exosomal FECR1 circRNA is a novel oncogenic driver that promotes tumor metastasis via the miR584-ROCK1 pathway; it is highly expressed in SCLC tissues and is positively correlated with lymph node metastasis [115]. Additionally, Chen et al. found that the PTEN/PI3K/AKT pathway is used by tumor-derived exosomal circFARSA to polarize M2 macrophages and promote NSCLC metastasis [116]. Moreover, using TGF-β as a model, Wang et al. demonstrated that circPTK2 suppresses TGF-β induced EMT and metastasis in NSCLC by regulating TIF1 [97]. Overexpression of circPTK2 may offer a treatment option for advanced non-small cell lung cancer and illuminate a novel approach by which circRNA regulates TGF-β induced EMT and tumor metastasis. Circ_0000519, another oncogenic circRNAs, overexpression of circ_0000519 promoted metastasis by targeting miR-1258 in NSCLC. Meanwhile, circ_0000519 inhibition decreased cell metastasis by reducing cyclin D1, vimentin, and MMP-9 expression levels. CircRNA hsa_circ_0020123 promotes metastasis via sponging miR-144 to relieve ZEB1 and EZH2 from inhibition [94]. In vitro and in vivo, suppressing hsa_circ_0020123 decreased NSCLC development and metastasis.

Further, circRNAs bind to RBPs in non-small cell lung cancer, which then allows them to influence EMT, invasion, and metastasis. CircLARP4 is a La-related RNA-binding protein and inhibits cell proliferation and metastasis by regulating SMAD7 expression [117]. A worse prognosis is related to reduced expression of circLARP4 in NSCLC. Moreover, the capacity for SPCA1 cells to metastasize is inhibited by overexpression of the circLARP4 gene [118]. Another circRNA down-regulated in NSCLC that may prevent lymphatic metastasis is hsa_circ_0033155. Inhibition of tumor growth, colony formation, and migration occur after ectopic expression of hsa_circ_0033155 [119].

### Exosomal circRNAs and apoptosis

The development of LC is linked to circRNAs, which have been implicated in several cellular processes, including proliferation, growth, metastasis, aging, and apoptosis [120]. Exo-circRNAs that are increased in LC have been found in several studies to decrease the apoptotic process and enhance tumor growth by sponging miRNAs. Recently, Li Chuankui and his colleagues showed that exosomal circPLK1 upregulation enhances the proliferation of NSCLC via acting on the miRNA-1294/high mobility cluster protein A1 pathway and inhibits apoptotic cell death [121]. According to Yang et al., circRNA TUBA1C sponging miR-143-3p increased the progression of NSCLC [122]. Furthermore, they found that circTUBA1C silencing led to elevated levels of cleaved caspase-3 and Bax protein expression which makes increasing apoptosis. Additionally, hsa circ 0012673 circular RNA, through regulating the miR-320a/LIMK18521 pathway, promotes LC cell growth and invasion [91]. By targeting miR-320a and upregulating LIM domain kinase 1, circ 0012673 could reduce proliferation, motility, and EMT and increase apoptosis in LUAD cell lines upon knockdown [91]. Likewise, according to Ding et al., increased circ-MEMO1 levels boosted aerobic glycolysis, cell cycle progression, and proliferation while inhibiting LC cell death through the miR-101-3p/KRAS pathway and was associated with poor prognosis [123].

Several studies have revealed that circular RNAs that are overexpressed in LC make tumors grow by increasing the expression of Bcl-2 or decreasing the expression of Bax, which inhibits the process of apoptosis. For example, by sponging miR-195 and triggering Bcl-2, circVANGL1 overexpression was found to behave as an oncogene and suppress LC apoptosis [124]. Furthermore, inhibition of apoptosis in LC cells was achieved by has_circ_0109320's ability to upregulate Bcl-2, downregulate Bax, and cleave caspase 3 and by its ability to sponge miR-595, induce E2F transcription factor 7 expression [125]. According to Qin et al. work, circPVT1 facilitates the progression of NSCLC cells by suppressing apoptosis and modulating the miR-497/Bcl-2 pathway. They discovered that circPVT1 controls the miR-497/Bcl-2 pathway and inhibits cell death by sponging miR-497 [126].

Despite this, several circRNAs are downregulated in LC and appear to have an antagonistic role in LC growth by inhibiting the Wnt axis. For example, through downregulating Wnt/β-catenin signaling and elevating ITCH expression, circ-ITCH served as a sponge for the expression of oncogenic miR-7 and miR-214 [127]. Tian et al. also revealed that the hsa circ 0043256 serves as a miR-1252 sponge, allowing it to bind ITCH and interfere with the Wnt/β-catenin pathway. They found that cinnamaldehyde-treated LC cells increased circ 0043256, which decreased cell growth and triggered apoptosis through ITCH in LUAD cell lines [128].

In contrast, the expression of circNOL10 was shown to be suppressed in LC, and it was also shown to promote apoptosis, which reduced LC proliferation in both in vivo and in vitro studies [129]. The molecular mechanism by which circNOL10 influenced SCML1's regulation of the human polypeptide family was the inhibition of transcription factor ubiquitination. Ultimately, circNOL10 induced cell death by upregulating the expression of Bax and caspase-9 while downregulating Bcl-2 expression [129]. Through interactions with members of the Bcl-2 family, circRNAs were found to regulate apoptosis in lung cancer. This finding opens the new approach for the development of targeted therapies.

### Exosomal circRNAs modulate drug resistance

Drug resistance is a significant concern in the management of cancer patients. Cancer cells can show resistance to treatment in a number of ways. Exosomes have gained universal attention as a novel therapeutic to treat cancer [130, 131]. Importantly, exosomes deliver non-coding RNAs (including circRNAs) and proteins linked with multi-drug resistance (MDR) to target cells [132]. Two MDR phenotypes exist. The first is the fundamental chemoresistance that predated medication exposure. However, the other is acquired resistance, which develops after extensive treatment [133]. Acquired MDR often develops during clinical cancer therapy and is a significant barrier to effectively inhibiting metastasis and cell proliferation, leading to a poor prognosis and short overall survival [134]. In addition, exosomes send functional P-glycoprotein to drug-sensitive recipient cells. This protein is a crucial part of the signaling pathways that help drug-sensitive recipient cells become resistant to drugs [135].

Numerous studies have found that circRNAs have a regulatory function in the resistance to cancers. For instance, lung adenocarcinoma (LAD) patients with high circPVT1 expression are less likely to respond to cisplatin and pemetrexed. CircPVT1 also leads to treatment resistance against these drugs by targeting the miR-145-5p/ABCC1 pathway [136]. Furthermore, Cao et al. found that inhibiting circ-PVT1 through the miR-429/FOXK1 signaling axis slowed LC growth and increased sensitivity to cisplatin [137]. Similarly, the lung cancer cell line circular RNA CDR1-AS promotes resistance to cisplatin and pemetrexed via activating the EGFR signaling pathway [138]. Additionally, the production of PD-L1 exosomes by NSCLC cells increased cell stemness, which in turn made tumor cells more resistant to cisplatin. By inhibiting PD-L1, chemoresistant tumor cells could be more sensitive to chemotherapy drugs such as cisplatin [139].

Recently, circRNAs that are increased in NSCLC have been identified to increase cisplatin resistance by promoting the expression of STAT3. For instance, circ 0076305 targeted miR-296-5p to actively modulate cisplatin resistance by overexpressing STAT3 in NSCLC [140]. Likewise, in LC cells, circAKT3 inhibited glycolysis and cisplatin resistance by controlling the miR-516b-5p/STAT3 pathway [141]. Meanwhile, Ma et al. observed that hsa_circRNA_0002130 had a high level of expression in the serum exosomes of osimertinib-resistant LC patients and osimertinib-resistant LC cells [142]. Accordingly, it has been hypothesized that circRNAs are critically involved in LC resistance pathways. Nevertheless, additional investigations will be needed to study those pathways that are triggered by exo-circRNAs in cancer patients.

## Therapeutic potential of exo-circRNAs

Exosomes are a promising therapeutic tool for many diseases because of their practical ability to transport small molecules between cells [143]. They may also be useful as biomarkers in a variety of diseases via modulating cell communications [144]. Due to their unique properties, such as their nano size, double lipid membrane, ability to act as multiple carriers, strong histocompatibility [145], high bioavailability [146], low cytotoxicity, and immunogenicity [147], exosomes can be used to deliver therapeutics to cancer cells. Furthermore, surface receptors make it easier for exosomes to target tumor cells and have less of a negative effect on healthy tissue [148].

Recent advancements in RNA-based therapies and altered RNA expression in cancers offer promising therapeutic strategies [149–151]. A new method is to develop synthesized circRNAs with high-affinity domains for specific oncogenic proteins, mRNAs, lncRNAs, and miRNAs that might be delivered exogenously to restore the cell's normal signaling pathway and inhibit tumor progression [152, 153] (Fig. 7). Additionally, exosomes, which are thought to be circRNA transporters, may be able to increase the number of circRNAs in cancer cells [154]. This will probably make cancer less aggressive and may act as a biomarker.

The production of synthetic circRNA sequences that can inhibit oncogenic miRNAs has become a very effective way to treat cancer because it can reduce the effectiveness of cancer’s compensatory mechanisms. For example, Kristensen et al. found that hybrid circRNAs might target oncogenic miRNAs and oncoproteins of the same pathway [155].

Additionally, circRNAs can also be used as sponges for oncomiRs [156]. Their expression level is also considered a treatment approach, such as sponging miRNA-9 via circMTO1, which makes it possible for p21 expression and inhibits cancer progression [157]. Similarly, Liu et al. revealed that synthetic circRNA named scRNA21 acts as a miR-21 sponge to inhibit the proliferation of cancer cells [158].

Furthermore, circRNAs can target oncoproteins and leads to inhibit the proliferation of tumor cells. For instance, inhibiting the Wnt/β-catenin axis with circular RNA-ITCH could also be used to treat different types of cancer [15]. Molecular analysis showed that oncogenic miR-7 and miR-214 were found to behave as a sponge for circRNA-ITCH, which increased ITCH expression and consequently reduced Wnt/β-catenin signaling in LC [127]. Likewise, by attaching to cell cycle proteins CDK2 and p21, circ-Foxo3 suppressed cell cycle progression when it was overexpressed [159].

Other circRNAs sponge miRNAs and mRNAs also proposed as a therapeutic option. The relevant mRNA expression in physiologic processes and pathological mechanisms was controlled by cross-talk between circRNAs and miRNAs [160]. The relative processes of interaction between circRNAs, miRNAs, and mRNAs are still being argued. However, two types of strategies have been described: (1) circRNAs sponge microRNAs, such as circHMCU can sponge the let-7 family and lead to cancer development and metastasis [161]. (2) Circular RNA is mediated by miRNAs. For example, in an Ago2-slicer-dependent manner, miR-671 cleaves a circular antisense transcript of the Cerebellar Degeneration-Related protein 1 locus (CDR1) [49]. CDR1 mRNA levels decreased due to circular antisense downregulation, even if heterochromatin does not occur.

In another way, some circRNAs are upregulated in malignant cells and can sponge tumor suppressor miRNAs, such as circGFRA1 and miR-34a [162]; and circUBAP2 and miR-143 [163], could be subjected to inhibition as a strategic way in cancer therapeutics. Furthermore, circRNA-MYLK acts as a ceRNA by binding miR-29a and facilitating the production of VEGFA [164]. The treatment of cancer has also been proposed for the silencing of this circRNA. The above studies consider that exosomal cirRNA-modulating may have potential applications in cancer therapies.

## Conclusion

Exosomes originating from LC cells, known as Lung cancer cell derived-exosomes (LCCDEs), play a role in the progression of LC. Exosomes, the smallest vesicle, deliver important cargo such as nucleic acids, lipids, and proteins. These molecules perform critical functions in cell-to-cell communication and are identified as promising markers for their diagnostic properties.

Exo-circRNAs are enriched in tumors and, with multiple configurations, have also recently received interest in their crucial function in LC carcinogenesis. It acts as sponge for microRNAs, binds to proteins, and interacts with the tumor microenvironment (TME). In addition, exo-circRNAs can be used in early diagnosis, therapeutic response, exosome drug-delivery design for target therapy, and prognosis.

Although the future holds great promise, various challenges should be overcome. Despite ongoing studies, several open concerns remain about the clinical use of mRNAs and exosomal circRNAs. From our perspective, exo-circRNAs will be one of the most hotly debated topics in the future, and further studies will be required to verify their clinical applications.

## Data Availability

The analyzed data sets generated during the study are available from the corresponding author on reasonable request.

## Abbreviations

LC: Lung Cancer
CircRNA: Circular RNA
TDEs: Tumor-derived exosomes
exo-circRNAs: Exosomal circular RNA
TME: Tumor microenvironment
MVB: Multivesicular body
ILVs: Intraluminal vesicles
ESCRT: Endosomal-sorting complex that is required for transport
VPS4: Vacuolar protein sorting-associated protein4
TSG101: Tumor susceptibility gene 101
ALIX: ALG-2-interacting protein X
ESCRT: Endosomal sorting complex required for transport
CHMP4: Charged multivesicular body protein 4a
RBPs: RNA-binding proteins
DHX9: DExH-box helicase 9
FUS: Fused in sarcoma
ADAR1: RNA-specific adenosine deaminase 1
NF90/NF110: Nuclear factor 90/110
MBL: Mannose‐binding lectin
QKI: KH domain containing RNA Binding
LUAD: Lung adenocarcinoma
WNT: Wingless-related integration site
PLAGL2: Pleomorphic adenoma gene like-2
EIciRNAs: Exon–intron circRNAs
Pol II: Polymerase II
U1 snRNA: U1 spliceosomal RNA
lncRNAs: Long non-coding RNAs
IRES: Internal ribosome entry site
UTR: Untranslated region
ORFs: Open reading frames
AUG: The codon for Methionine
hESCs: Human embryonic stem cells
Ago2: Argonaute 2
rRNA: Ribosomal RNA
TNM: Tumor, nodes, and metastases
FASN: Fatty acid synthase
STAT3: Signal transducers and activators of transcription 3
NSCLC: Non-small cell lung cancer
KPNA4: Karyopherin subunit alpha-4
E2F5: E2F Transcription Factor 5
EZH2: Enhancer of zeste 2 polycomb repressive complex 2 subunit
SLC1A5: Solute carrier family 1 member 5
ZEB1: Zinc finger E-box-binding homeobox 1
TIF1-y: Transcription intermediary factor 1-gamma
RASSF8: Ras association domain family nember 8
TGF-β: Transforming growth factor beta
KRAS: Kirsten rat sarcoma virus
Bcl-2: B-cell lymphoma 2
E2F: Family of transcription factors
SCML1's: Scm polycomb group protein like 1
MDR: Multiple drug resistance
LAD: Lamina-associated domains
ABCC1: ATP binding cassette subfamily C member 1
FOXK1: Forkhead box K1
EGFR: Epidermal growth factor receptor
PD-L1: Programmed death-ligand 1
FOXO3: Factor forkhead box O-3
CDR1: Cerebellar degeneration related protein 1
LCCDEs: Linear constant-coefficient difference equation
